# Supplementary material for: Modeling glioblastoma heterogeneity as a dynamic network of cell states
Source: Mol Syst Biol. 2021 Sep 16;17(9):e10105. doi: 10.15252/msb.202010105 (PMC8444284; doi:10.15252/msb.202010105)
Supplement: Supplementary file 6 — Source Data for Figure 5 [file MSB-17-e10105-s004.zip › Figure5A_sourcedata/GSEA_3017/hallmarks_stateD.GseaPreranked.1621934542601/gsea_report_for_na_pos_1621934542601.html]

Report for na\_pos 1621934542601 [GSEA]

| GS  follow link to MSigDB | GS DETAILS | SIZE | ES | NES | NOM p-val | FDR q-val | FWER p-val | RANK AT MAX | LEADING EDGE || 1 | HALLMARK\_FATTY\_ACID\_METABOLISM | Details ... | 19 | 0.24 | 1.27 | 0.154 | 0.327 | 0.490 | 216 | tags=42%, list=16%, signal=50% |
| 2 | HALLMARK\_ANDROGEN\_RESPONSE | Details ... | 17 | 0.16 | 0.78 | 0.775 | 0.769 | 0.959 | 1109 | tags=100%, list=84%, signal=615% |
Table: Gene sets enriched in phenotype **na **[plain text format]****
